# Novel protein contact points among TP53 and minichromosome maintenance complex proteins 2, 3, and 5

**DOI:** 10.1002/cam4.4805

**Published:** 2022-05-14

**Authors:** Stephanie Schaefer‐Ramadan, Jovana Aleksic, Nayra M. Al‐Thani, Joel A. Malek

**Affiliations:** ^1^ Department of Genetic Medicine Weill Cornell Medicine in Qatar Doha Qatar

**Keywords:** cancer, DNA helicase, DNA replication, protein interactions, therapeutic targets, TP53

## Abstract

**Objective:**

Identify protein contact points between TP53 and minichromosome maintenance (MCM) complex proteins 2, 3, and 5 with high resolution allowing for potential novel Cancer drug design.

**Methods:**

A next‐generation sequencing‐based protein–protein interaction method developed in our laboratory called AVA‐Seq was applied to a gold‐standard human protein interaction set. Proteins including TP53, MCM2, MCM3, MCM5, HSP90AA1, PCNA, NOD1, and others were sheared and ligated into the AVA‐Seq system. Protein–protein interactions were then identified in both mild and stringent selective conditions.

**Results:**

Known interactions among MCM2, MCM3, and MCM5 were identified with the AVA‐Seq system. The interacting regions detected between these three proteins overlap with the structural data of the MCM complex, and novel domains were identified with high resolution determined by multiple overlapping fragments. Fragments of wild type TP53 were shown to interact with MCM2, MCM3, and MCM5, and details on the location of the interactions were provided. Finally, a mini‐network of known and novel cancer protein interactions was provided, which could have implications for fundamental changes in multiple cancers.

**Conclusion:**

We provide a high‐resolution mini‐interactome that could direct novel drug targets and implicate possible effects of specific cancer mutations.

## Graphical abstract

Using a next‐generation sequencing‐based protein‐protein interaction method, we identified interactions among MCM2, MCM3, and MCM5. While many of the interacting regions we detected overlap with existing structural data of the complex, we also recovered novel domains with higher resolution. Additionally, we were also able to identify, for the first time, that wildtype TP53 interacts with MCM2, MCM3, and MCM5 and provide the location of the interactions.

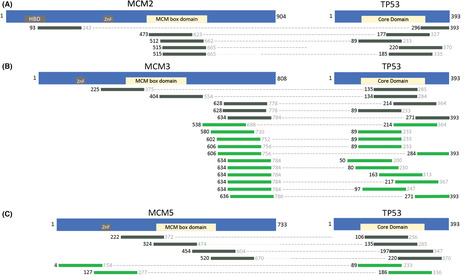

## 1 INTRODUCTION

The minichromosome maintenance (MCM) proteins 2–7 make up the eukaryotic helicase motor and are essential for DNA replication and repair.^1^ As evidence of their functional and stochiometric importance, these six proteins are evolutionarily highly conserved, and their expression needs to be carefully balanced for normal, controlled cell cycle progression.^2^ Deregulation of the MCM complex could lead to tumorigenesis.^3^, ^4^, ^5^ MCM proteins are highly expressed in malignant human cancerous and pre‐cancerous cells.^3^ A non‐exhaustive list suggests that MCM2,^6^, ^7^, ^8^, ^9^, ^10^, ^11^ MCM3,^9^, ^10^, ^12^ MCM4,^9^, ^10^, ^13^, ^14^ MCM5,^10^, ^14^, ^15^ MCM6,^10^, ^16^ and MCM7^6^, ^10^, ^12^ could be powerful diagnostic and prognosis markers in numerous cancer types (also see recent review^17^).

While the MCM complex 2–7 is essential, several studies suggest the individual proteins may function outside the formation of a complex, as demonstrated by mutational analysis in yeast^18^ and other enzymatic studies with MCM5,^19^ MCM3,^20^, ^21^, ^22^ and MCM2.^23^, ^24^ These six MCM proteins share homologous domain organization with some apparent differences.^25^ For example, MCM3 and MCM5 have flexible winged‐helix domains attached to AAA+ domain at their C‐termini which are disordered in the recent cryo‐EM structure.^26^, ^27^ In contrast, MCM2 has a disordered N‐terminal extension which is absent in both MCM3 and MCM5. The importance of these flexible regions for protein interactions and recruiting should not be underestimated, as highlighted in a recent review by Zhai and colleagues.^25^

TP53 was identified in the 1970s.^28^ In 1989, Baker and colleagues discovered essential properties of the TP53 gene in colorectal carcinoma.^29^ Shortly after this breakthrough study, researchers demonstrated wild type TP53 is a tumor‐suppressing protein^30^, ^31^, ^32^ involved in cellular functions such as apoptosis, cell cycle control, and cell differentiation. The C‐terminal region of TP53 (residues 374–388) is highly basic and intrinsically disordered. It can adopt multiple confirmations depending on the substrate, meaning identical residues are used for different protein–protein interaction interfaces.^33^ For example, the N‐terminal transcriptional activation domain (TAD domain) of TP53 is completely disordered in solution, but upon binding a substrate, it folds.^33^ Work by Weinberg et al. shows that the importance protein–protein interactions stemming from the TP53 core domain are for the cooperative binding of p53 to DNA.^34^

TP53 is the most frequently mutated human cancer gene^35^ and is characterized by many naturally occurring mutations. Missense mutations in TP53 are most prevalent in cancer with specific mutations being associated with worse survival prognosis.^36^, ^37^, ^38^, ^39^, ^40^, ^41^, ^42^, ^43^ Most cancer‐related missense mutations occur in the core domain (DNA binding domain) of TP53 and result in a loss of function.^44^ These mutant TP53 proteins form mixed tetramers with wild type producing dominant‐negative effects.^44^ Interestingly, “hot spot” mutations to the TP53 core domain can cause a gain‐of‐function (GOF) phenotype^44^, ^45^ and can indicate resistance to drug therapies.^35^ Indeed, the GOF mutations in TP53 are underexplored in cancers regarding loss of function and might present an exciting opportunity for protein interaction studies if these mutations result in stronger constitutive or novel interactions compared to wild type.

The all‐vs‐all sequencing (AVA‐Seq) method was recently used with a human interaction set to establish how well the AVA‐Seq process could recover binary interactions.^46^ This method combines bacterial two‐hybrid screening with a next‐generation DNA sequencing readout of growth changes of the host cells signifying potential protein–protein interactions. Our previous study ^46^ and the work of others ^47^, ^48^, ^49^, ^50^, ^51^ indicated interactions among MCM complex proteins and TP53 (also see Table S5). The interactions between MCM2|MCM5,^1^, ^19^, ^52^, ^53^, ^54^ MCM2|MCM3^52^, ^53^, ^54^, ^55^ and MCM5|MCM3^1^, ^19^, ^52^, ^53^, ^54^, ^55^, ^56^ are well documented. However, the interaction between TP53 and the MCM2‐7 complex was less clear. For example, the GOF TP53 mutant R273H directly interacts with MCM2, MCM4, and MCM5, while wild type TP53 does not.^47^, ^48^, ^49^ Therefore, our goal for this study was to identify potentially novel cancer‐relevant protein interactions among TP53, MCM2, MCM3, and MCM5. We identify where these proteins interact by pinpointing protein–protein fragment pairings which are supported by multiple fusion orientations, overlapping fragments, and increasing strength of the competitive inhibitor, 3‐AT. Additionally, our data support that our AVA‐Seq method can detect weak/transient protein–protein interactions. This manuscript aims to provide clearer targets for disrupting these interactions when they become dysregulated.

## 2 MATERIALS AND METHODS

### 2.1 Materials

Materials and methods used were as described previously.^46^ Briefly, the open reading frames for the human reference protein interaction set were supplied in Gateway vectors^46^ (hsPRS‐v2; hsRRS‐v2). The TP53 construct in the hsPRS‐v2 contains two mutations (isoform 1 identifier: P04637‐1 with mutations P72R and P278A). The Validation Reporter bacteria cell line was transformed with the pAVA plasmids containing the fragmented fusion proteins to allow for interaction screening using minimal media in the presence or absence of the competitive HIS3 inhibitor, 3‐amino‐1,2,4‐triazole (3‐AT). AVA‐Seq plasmids were deposited previously and are available for purchase from Addgene.^57^ Illumina library generation utilized NEBNext Ultra II DNA Library Prep Kit (E7645L) and quantification materials (KAPA Illumina Library Quantification KK4824) as reported previously.^46^, ^57^

### 2.2 AVA‐Seq method

The all‐vs‐all sequencing (AVA‐Seq) method has been detailed previously.^46^, ^57^ Briefly, we have modified a bacterial two‐hybrid system to incorporate protein fragments on one plasmid, pAVA, in convergent orientation fused to either RNAp or λC1. First selected genes were PCR amplified, sheared, and then ligated into vectors, allowing for open reading frame (ORF) filtering. Fragments enriched for codon frame one are then amplified and ligated into the final pAVA plasmid followed by transformation of a modified *Escherichia coli* cell line allowing for protein interactions to survive in the presence of 3‐AT, a competitive HIS3 inhibitor. The novelty in this approach affords protein interactions to be mapped with a higher resolution in a high‐throughput manner; millions of protein fragment pairs can be screened simultaneously. The system also can titrate the interaction strength via the competitive inhibitor (3‐AT) concentration in the media. By increasing the 3‐AT concentration, the user can select for stronger protein–protein interactions. Here, the use of 2 and 5 mM 3‐AT concentrations allowed us to detect a range of interaction strengths.

### 2.3 Data collection

Data were collected from an Illumina NGS platform using standard library and sequencing chemistry methods for paired 150 bp read lengths per the manufacturers' recommended protocol. Fastq files of paired‐end sequences were aligned to a sequence database of the human protein interaction reference set and read counts for each fragment pair were normalized and scaled using methods as described previously.^46^ Read counts were statistically analyzed in the EdgeR package as previously described.^46^ Interacting fragment pairs were determined using cutoffs of Log_2_ fold change of the normalized read counts of greater than or equal to 1 and a False Discovery Rate of <10%.

### 2.4 Identifying protein contact points

Using recent structural data (PDB 6xtx), protein contact points between MCM proteins were identified using 5 Å distance^58^ in PyMOL and mapped to a linear schematic of the proteins. If applicable, other features such as a histone binding domain (HBD), zinc fingers (ZnF), MCM box domains, and disordered regions were identified for each protein with data provided in UniProt.org.

## 3 RESULTS

### 3.1 MCM2 and MCM5

**FIGURE 1:**
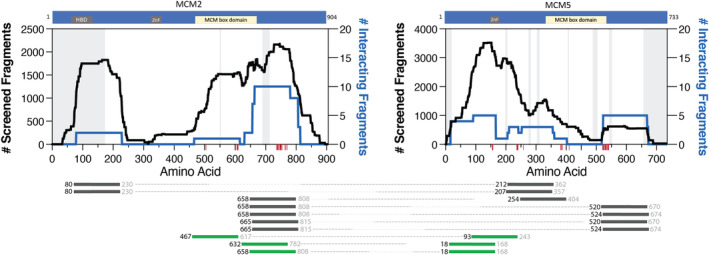
MCM2|MCM5 interacting fragment pairs. The schematics of the proteins on the top show the location of ZnF, histone binding domain (HBD), and MCM box domains. The black trace illustrates the noninteracting fragments (left y‐axis), and the blue trace plots the interacting fragments (right y‐axis) between the two proteins plotted against the amino acid numbering (x‐axis). Red ticks along the x‐axis identify protein contact points between the two proteins identified from PDB 6xtx. Disordered regions according to cryo‐EM structure PDB 6xtx are gray shaded regions on the graph. Below, fragments are identified by the starting residue (black color font), which was positively identified with sequencing data. The fragment lengths are approximately 150 amino acids in length. Therefore, the ending residue (gray color font) is an estimate. Dotted lines connect the interacting fragments between MCM2 and MCM5. The dark gray fragment pairs were recovered only in 2 mM 3‐AT, while the green fragment pairs were recovered in 5 mM or both 2 and 5 mM 3‐AT, indicating a stronger interaction between the fragment pairs. The order of the interacting fragment pairs (from top to bottom) is not significant

The AVA‐Seq method recovered 10 interacting fragment pairs between MCM2 and MCM5 (Figure 1). Three fragment pairs were recovered in both mild and robust selective conditions (Figure 1, green fragment pairs; Table S1).^46^ The MCM2 fragments interacting with MCM5 predominantly localize near the C‐terminal disordered region of MCM2, with consensus among the fragments being residues 665–782, approximately. Protein contact points were identified and illustrated as red ticks on x‐axis (Figure 1; see Materials and Methods).

The average fragment length tested in this study is 150 residues^46^ where the starting residue is identified during the sequencing step, but the end of the fragment is an estimate based on shearing and size selection. Therefore, the C‐terminus of MCM5 fragments (such as fragment MCM5:18) may contain a portion of the ZnF (which begins at residue 174) and a protein contact point. If these specific fragments extend past residue 174, all MCM5 fragments (in the MCM2|MCM5 interaction) would contain at least one protein contact point. Similarly, eight out of 10 MCM2 fragments include a protein contact point, with the remaining two fragments being localized to the N‐terminal disordered region of MCM2.

### 3.2 MCM2 and MCM3

**FIGURE 2:**
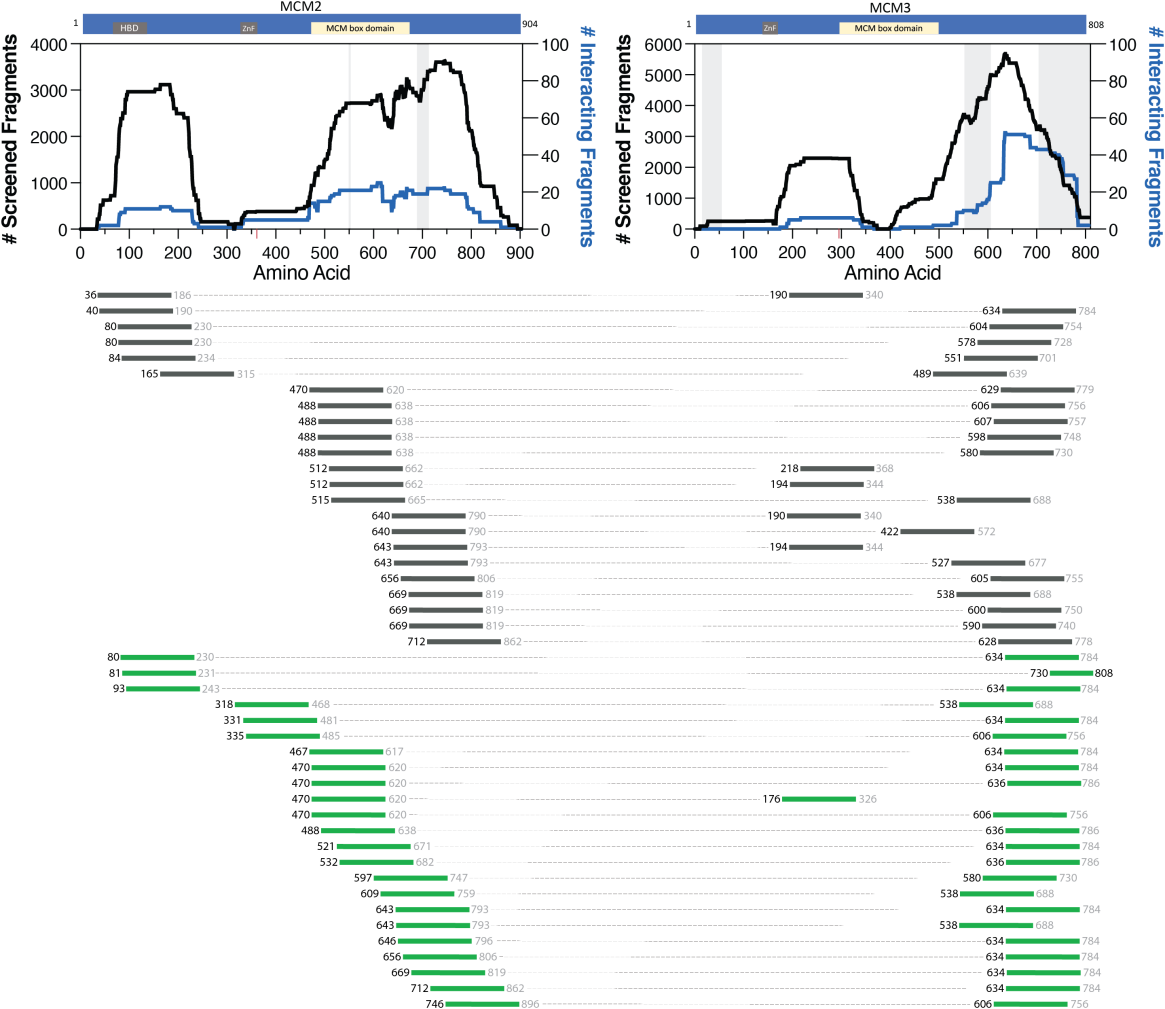
MCM2|MCM3 interacting fragment pairs. The schematics of the proteins on the top show the location of ZnF, histone binding domain (HBD), and MCM box domains. The black trace illustrates the noninteracting fragments (left y‐axis), and the blue trace plots the interacting fragments (right y‐axis) between the two proteins plotted against the amino acid numbering (x‐axis). Red ticks along the x‐axis identify protein contact points between the two proteins identified from PDB 6xtx. Disordered regions according to cryo‐EM structure PDB 6xtx are gray shaded regions on the graph. Below, fragments are identified by the starting residue (black color font), which was positively identified with sequencing data. The fragment lengths are approximately 150 amino acids in length. Therefore, the ending residue (gray color font) is an estimate. Fragments that start <150 amino acids from the C‐terminus have their ending residue in black as the fragment would stop at the C‐terminal stop codon. Dotted lines connect the interacting fragments between MCM2 and MCM3. The dark gray fragment pairs were recovered only in 2 mM 3‐AT. While the green fragment pairs were recovered in 5 mM or both 2 and 5 mM 3‐AT, indicating a stronger interaction between the fragment pairs. The order of the interacting fragment pairs (from top to bottom) is not significant

The MCM2|MCM3 interaction (Figure 2; Table S2) shows most MCM2 fragments interacting with the C‐terminal disordered region of MCM3. This C‐terminal localization is more apparent with fragment pairs recovered in 5 mM 3‐AT (Figure 2, green fragment pairs). The MCM2 fragments cover the span of the protein and are somewhat localized to the MCM domain and an N‐terminal disordered area. The red tick in Figure 2 shows a single protein contact point identified from the cryo‐EM structure (PDB 6xtx). Due to the apparent disordered nature of MCM2 and MCM3, it is impossible to determine if these are the only two residues involved in protein contact without further in vitro experiments (Figure 2, gray shaded area).

### 3.3 MCM5 and MCM3

**FIGURE 3:**
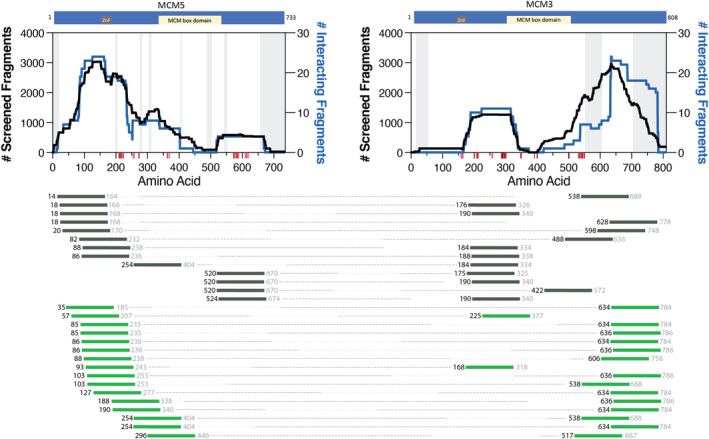
MCM5|MCM3 interacting fragment pairs. The schematics of the proteins on the top show the location of ZnF and MCM box domains. The black trace illustrates the noninteracting fragments (left y‐axis), and the blue trace plots the interacting fragments (right y‐axis) between the two proteins plotted against the amino acid numbering (x‐axis). Red ticks along the x‐axis identify protein contact points between the two proteins identified from PDB 6xtx. Disordered regions according to cryo‐EM structure PDB 6xtx are gray shaded regions on the graph. Below, fragments are identified by the starting residue (black color font), which was positively identified with sequencing data. The fragment lengths are approximately 150 amino acids in length. Therefore, the ending residue (gray color font) is an estimate. Dotted lines connect the interacting fragments between MCM5 and MCM3. The dark gray fragment pairs were recovered only in 2 mM 3‐AT. While the green fragment pairs were recovered in 5 mM or both 2 and 5 mM 3‐AT, indicating a stronger interaction between the fragment pairs. The order of the interacting fragment pairs (from top to bottom) is not significant

MCM5|MCM3 is a physiological interaction referenced in the literature and detected with our system. The interacting fragments between MCM5 and MCM3 and the enrichment in protein contact points were identified from the structure (PDB 6xtx) (Figure 3; Table S3). The fragment pairs in gray represent those recovered only in 2 mM 3‐AT conditions, while the green fragment pairs represent stronger interactions found in 5 mM or both 2 and 5 mM 3‐AT conditions. Of particular interest is the repeated localization of the interacting pairs which are identified by slightly shifted fragments. For example, MCM5:85 is partnered with two unique MCM3 fragments, MCM3:634 and MCM3:636. Moving just one amino acid toward the C‐terminus, MCM5:86 is partnered with these same MCM3:634 and MCM3:636 fragments. This provides both confirmation through repeated observation by different fragments and localizes the interaction with more resolution than traditional two‐hybrid interaction studies as they generally utilize full‐length proteins.

### 3.4 TP53 and the MCM complex proteins

**FIGURE 4:**
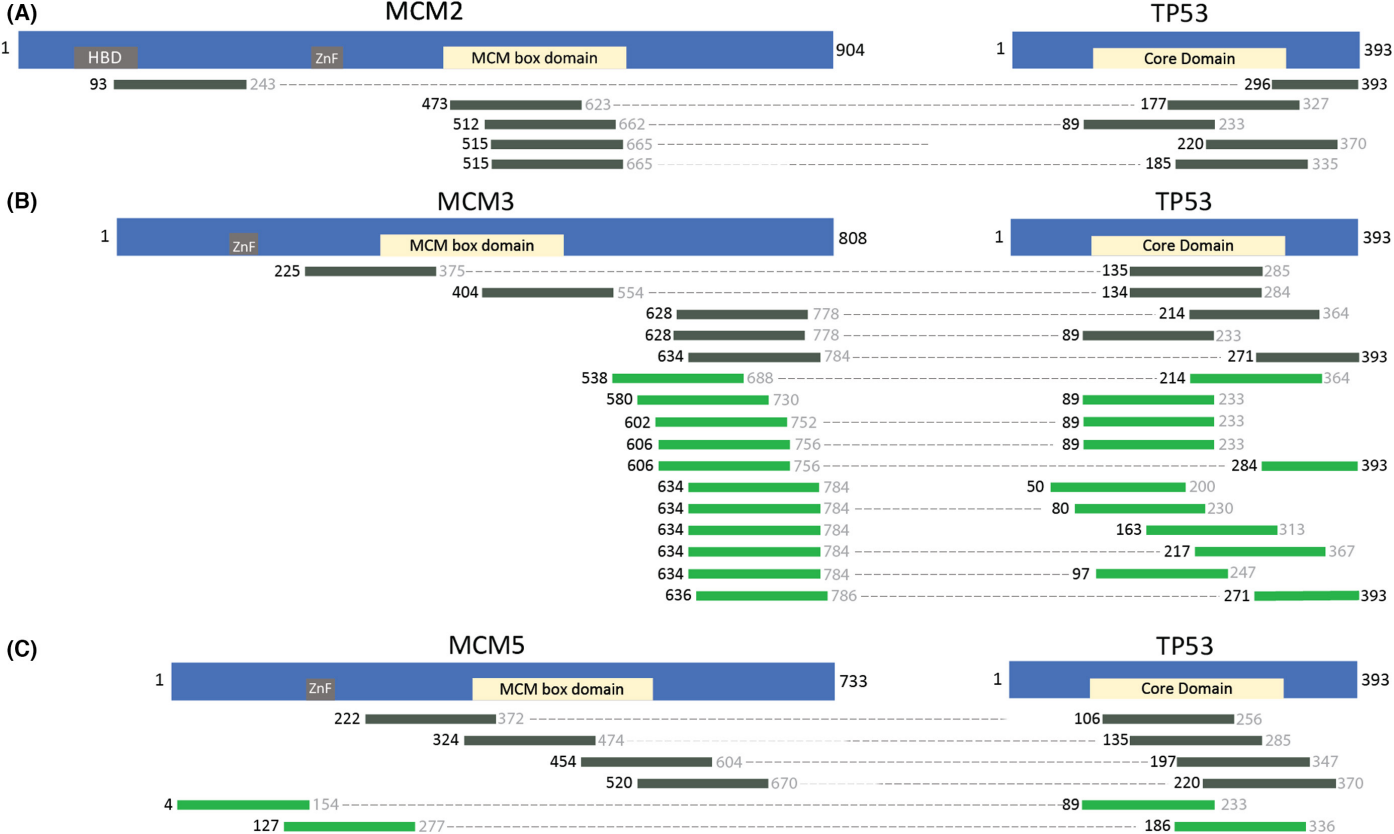
Interacting fragments of minichromosomal maintenance proteins with TP53. (A) Interacting fragment pairs of MCM2 and TP53, (B) Interacting fragment pairs of MCM3 and TP53, (C) Interacting fragment pairs of MCM5 and TP53. The schematics of the MCM proteins are the same as in Figures 1, 2, 3. The TP53 schematic highlights the Core Domain of the protein in yellow. Fragments are identified by the starting residue (black color font), which was positively identified with sequencing data. The fragment lengths are approximately 150 amino acids in length. Therefore, the ending residue (gray color font) is an estimate. Fragments that start <150 amino acids from the C‐terminus have their ending residue in black as the fragment would stop at the C‐terminal stop codon. Dotted lines connect the interacting fragments between MCM proteins and TP53. The dark gray fragment pairs were recovered only in 2 mM 3‐AT. While the green fragment pairs were recovered in 5 mM or 2 and 5 mM 3‐AT, indicating a more robust interaction. The order of the interacting fragment pairs (from top to bottom) is not significant. For more details on the interacting fragment pairs, see Table S4

All five MCM2|TP53 interacting fragment pairs were recovered in the mild selective pressure conditions (Table S4, 2 mM 3‐AT). Most of these MCM2 interacting fragments localize to the MCM box domain (Figure 4A, residues 473–679 of MCM2) except for one fragment (MCM2:93|TP53:296) in which the MCM2:93 fragment is localized to the N‐terminal disordered region and histone binding domain (also see Discussion). Two of the five MCM2 fragments are paired with a wild type TP53 fragment (Table S4).

Interacting fragment pairs for TP53|MCM3 are predominantly localized to the C‐terminal disordered region of MCM3 (Figure 4B, residues 662–739 of MCM3; Table S4) as seen with the MCM2|MCM3 and MCM5|MCM3 interactions. Two additional TP53|MCM3 fragments localize to the MCM box domain of MCM3 (residues 295–502 of MCM3; interacting fragment pairs TP53:135|MCM3:225 and TP53:134|MCM3:404) and the TP53 core domain. This is not surprising as the core domain of TP53 plays a vital role in stabilizing protein–protein interactions.^59^

TP53|MCM5 interacting fragment pairs recovered with this study are shown in Figure 4C. Two TP53|MCM5 fragment pairs interact in the strong competitive inhibitor (Table S4, 5 mM 3‐AT). All the TP53 fragments align to the core domain of TP53, with some overlap in the C‐terminal disordered regions. This is not surprising given the size of TP53 and the average fragment length. Additionally, there are MCM5 fragments that fully or partially overlap with the MCM box domain, while others are closer to the N‐terminal disordered part of the protein.

Table S4 lists interacting fragments between TP53 and MCM2, MCM3, or MCM5. Of particular interest are seven wild type TP53 fragments (noted with an *) that interact in the most robust selective conditions (5 mM 3‐AT). Convincingly, the wild type TP53:89 fragment is paired with fragments from MCM2, MCM3, and MCM5.

### 3.5 Coverage of interaction space

**FIGURE 5:**
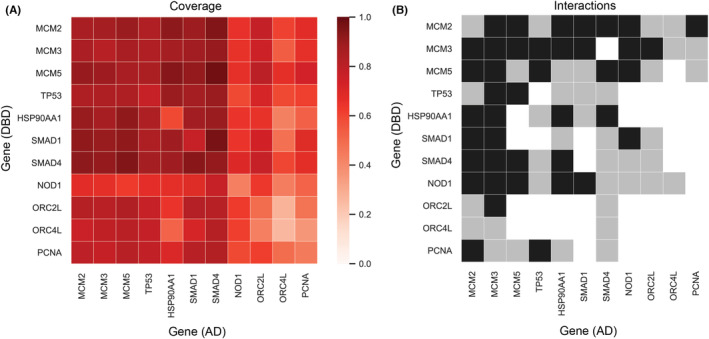
Protein coverage and interactions recovered. (A) Heatmap of protein coverage of protein pairs recovered in 0, 2, or 5 mM 3‐AT. The scale represents depth or overlap of sequence coverage for a protein pair. Protein pairs with poor sequence coverage (meaning fragment combinations between the two proteins do not adequately cover all possible amino acid pairings) are closer to 0.0 (light red) and proteins with high sequence coverage are closer to 1.0 (dark red). (B) Protein interactions recovered in 2 mM exclusively (gray) or 2 mM and 5 mM 3‐AT (black) are represented on the interaction map. Note: no protein–protein interactions from this mini‐interactome were recovered in only 5 mM 3‐AT conditions. However, there may be specific protein fragment pairs from interactions that are found only in 5 mM 3‐AT (see Tables S1–S4)

Figure 5A shows a heat map of the screened protein interaction area. In this panel, the dark red color indicates high protein sequence coverage of the two protein fragments paired together in the pAVA plasmid, meaning one or more unique fragments cover the primary sequence of both proteins. The lighter red color indicates one or both proteins have fragments localized to a portion of the protein, meaning some of the interaction areas between the two proteins have lower or no coverage. Figure 5B shows the enrichment of interactions in the presence of selective media containing 3‐AT. AVA‐Seq has both orientations (DBD‐ or AD‐fusion represented by the y‐ and x‐axis, respectively) built into the method, increasing the chance of capturing interactions that might otherwise be missed. For example, the MCM3|SMAD4 interaction has coverage >80% in both orientations (Figure 5A), but the interaction was detected in only one orientation (Figure 5B). It is important to mention that full coverage of smaller proteins is easier to accomplish as they might require as few as two fragments. At the same time, larger proteins would need more fragments to cover the primary amino acid sequence. Coverage of some proteins, especially on the N‐ and C‐termini, may have been underrepresented due to likely bias from the open reading frame (ORF) filtering technique.^46^

### 3.6 Known and novel interactions recovered

**FIGURE 6:**
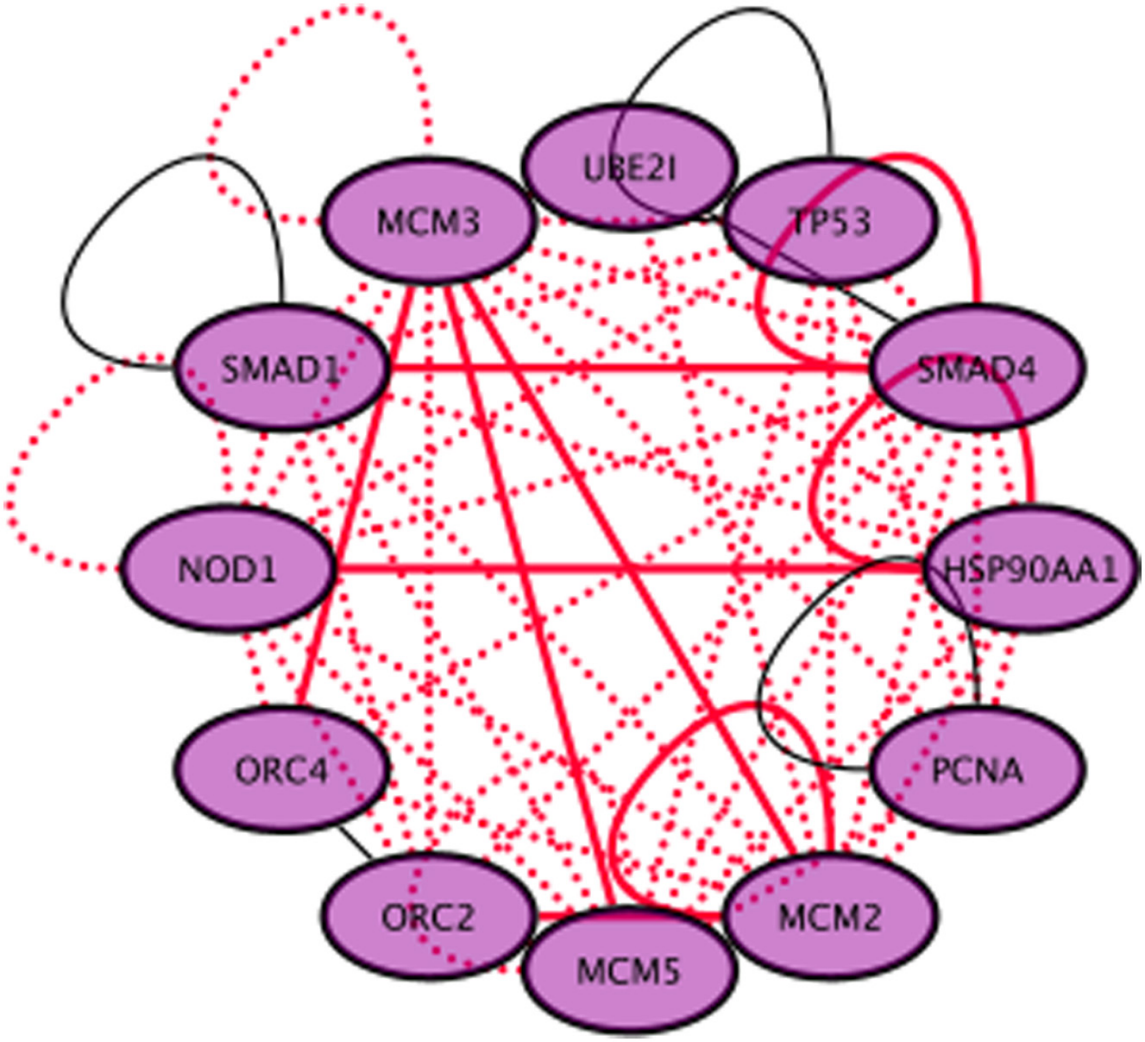
Mini‐network of protein–protein interactions involving MCM2, MCM3, MCM5, TP53, and others. Known interactions are shown as solid lines, with red‐solid interactions recovered with AVA‐Seq and black‐solid interactions not recovered with AVA‐Seq. Dashed red lines are novel interactions recovered with AVA‐Seq using this small pool of interacting proteins. Data for known interactions were obtained from HiUnion^61^

Interactions such as ORC2|MCM2,^52^, ^54^ ORC2|MCM5,^54^ ORC2|MCM3,^56^ MCM2|ORC4,^54^ MCM3|ORC4,^52^, ^54^ MCM3|HSP90AA1,^60^ MCM2|HSP90AA1,^1^, ^60^ MCM5|HSP90AA1^1^, ^60^ were reported in the literature and detected using the AVA‐Seq method.^46^ These interactions, along with other potentially novel biological interactions are illustrated as an interaction network in Figure 6. These novel protein–protein interactions (red dashed line in Figure 6) include SMAD1|MCM3, SMAD1|MCM2, SMAD4|MCM3, SMAD4|MCM2 and SMAD4|MCM5, MCM2|NOD1, MCM3|NOD1, MCM2|PCNA, MCM3|PCNA, and MCM5|PCNA. Although literature demonstrating direct interactions between these proteins were not found, it is not outlandish to think these proteins interact as the tested protein interaction set is enriched with cancer proteins of similar pathways or function. For example, the MCM proteins and PCNA are prolific markers for breast cancer and are participants in DNA replication.^27^

## 4 DISCUSSION

This study provided an opportunity to identify physiologically relevant interacting fragments using the AVA‐Seq method. This was done by overlapping interacting fragment pairs between MCM2, MCM3, and MCM5 with cryo‐EM protein contact points (Figures 1, 2, 3). However, not all interacting fragments contain a protein contact point(s). For example, in MCM2, a fragment starting at amino acid residue 80 (represented by the nomenclature MCM2:80) appears to localize to the N‐terminal disordered regions of the protein. It does not seem to be associated with a protein contact point from the cryo‐EM structure. It would be interesting to investigate further a correlation between fragments involved with a protein contact point being more likely to be recovered in more robust interaction screening conditions (i.e., 5 mM 3‐AT).

MCM2 in Figure 1 shows a clearer resolution interaction between MCM2 and MCM5. By this we mean the interaction(s) between the proteins are more isolated to one region of the protein and/or multiple fragments overlap, increasing confidence that a particular region is involved in a protein–protein interaction. We have observed similar resolution previously.^46^ At the same time, we note that some of the interactions highlighted in this manuscript do not show single contact resolution. Instead, they indicate proteins that likely have multiple contact points across the full protein as evidenced by interacting fragments spanning large sections of the proteins. This may be the case with the MCM2, MCM3, and MCM5 proteins, as they are known to form a protein complex and have protein contact points distributed throughout the length of the proteins. It is important to remember that two contact points that appear far apart linearly may be in proximity when considering the 3D structure.^46^ If this is the case, some resolution may be lost or more challenging to interpret especially in the absence of a 3D structure.

Interacting fragments near the C‐terminus of MCM3 are enriched in the more robust selective conditions (green fragment pairs) in both the MCM2|MCM3 interaction (Figure 2) and the MCM5|MCM3 interaction (Figure 3). Some of the interacting pairs in 5 mM 3‐AT give resolution of the interaction region in tiny increments (i.e., many fragments with similar starting points), attesting to the significance of the fragment locations and method sensitivity. The C‐terminal fragment clustering could indicate a role for the C‐terminal disordered region of MCM3 in stronger protein–protein interactions. There is a similar enrichment in the C‐terminal region with MCM3 fragments paired with TP53 protein (Figure 4B).

Not surprisingly, interacting fragment pairs appear to localize differently when comparing interactions recovered exclusively in the 2 mM 3‐AT condition (gray colored fragments) and fragments recovered in both 2 and 5 mM or exclusively in 5 mM conditions (green colored fragments) in Figures 1, 2, 3, 4. Visualizing this change has added value by allowing the user to make predictions based on fragment location and binding strength. The apparent differences in binding preference are pronounced in the MCM5|MCM3 interaction. For example, when comparing the MCM5|MCM2 (Figure 1) and MCM5|MCM3 (Figure 3) interactions, it is clear that the MCM5 residues adjacent to the MCM box domain (inclusive of 520–650) are important for interactions between these proteins under mild (2 mM 3‐AT) selective pressure. Although the potential physiological implications need to be investigated further, it is clear that these data go beyond the binary protein–protein interaction data from traditional two‐hybrid assays allowing users of the AVA‐Seq system to isolate protein contact points under several conditions.

The C‐terminal region of TP53 has at least 40 different binding partners and utilizes the intrinsically “disordered region for mediating and modulating” PPIs.^62^ The N‐ and C‐termini of TP53 have several predicted linear motifs^63^ that mediate weak interactions (K_d_ = ~1–150 μM) but show high specificity.^64^ The transient nature of these linear motifs allows for rapid response to changing environments (i.e., signaling proteins).^64^, ^65^ The number of TP53 interacting fragments from our study that localize or contain a portion of the disordered sequence is interesting—again, adding to the possibility that our fragmented AVA‐Seq approach may preferentially recover interactions localized to disordered or flexible regions of proteins.^46^ We plan to explore this potential correlation in more detail in the future as we believe the AVA‐Seq method could give insight into interaction regions of disordered proteins, which are inherently challenging to crystalize due to their flexibility, and other protein–protein interactions, which are more transient.

With respect to transient interactions––work by others showed strong interactions between certain MCM proteins and GOF TP53 mutants but failed to detect an interaction with wild type until in vivo methods capable of detecting transient interactions were used. We have recovered many interacting fragment pairs between the MCM2, MCM3, and MCM5 proteins with wild type TP53. Interestingly, there were no interacting fragment pairs between MCM2|TP53 in the 5 mM 3‐AT conditions. This could indicate a more transient interaction between MCM2|TP53 when compared to the TP53 interactions with MCM3 or MCM5. It would be of interest to further investigate the preferences of TP53 binding with regards to the MCM2‐7 proteins especially given their similar domain structures.

We observe interactions of the MCM proteins with TP53 localizing outside of the MCM box domains in 5 mM 3‐AT, while the fragment pairs recovered in only 2 mM seem to have a preference to contain the MCM box domains (Figure 4). This is not unlike the change in fragment enrichment of the MCM interacting proteins as a function of 3‐AT concentration (Figures 1, 2, 3). These changes may indicate possible drug target locations between TP53 and MCM proteins which reside outside of the ATP binding sites of the MCM proteins as specific interaction sites appear to be more robust (i.e., bind tighter). There have already been several small molecules identified to target MCM proteins via downregulation of a single component or enzymatic inhibition of the entire complex.^27^ Given the strong functional correlation between MCM complex proteins and cancer, the need for further knowledge of the MCM interactome is of extreme importance to expand the cancer therapeutics toolbox.^2^

There are multiple lines of evidence including in vivo and in vitro experiments that both mutant TP53 and wild type TP53 interact with the MCM proteins (Table S5). Additionally, wild type TP53 interactions are weaker/transient than the GOF mutant TP53 interactions and, therefore more challenging to detect. Work by others demonstrated MCM2 interacted with both wild type TP53 and TP53 R273H.^50^ Additionally, they showed TP53 R273H Δ381‐388 (removal of C‐terminal residues) do not seem essential for interaction with MCM2 while deletion of a larger portion (TP53 R273H Δ347‐393) reduced interaction with MCM2.^50^ Interestingly, TP53 fragments from Figure 4A, which may not contain the 347–393 region (TP53:177, TP53:89, TP53:185), interact with MCM2 fragments localized to the MCM box domain. The shortest TP53:296 fragment is paired with an MCM2 fragment that localizes differently than the others in Figure 4A. However, a comparison between the Annor et al. study and this study might not be reasonable as our TP53 construct contains a different TP53 mutation. Their work and ours predict the TP53 interaction with MCM2 to be a weak interaction.^50^

A double positive association exists between mutant TP53 R175H and all MCM2‐7 complex proteins in both the cytosol and nucleus as determined via proteomic enrichment analysis.^48^ Low levels of wild type TP53 were detected interacting with MCM2 in MCF‐7 cells^48^ and further confirmed with proximity ligation assay. Bargonetti and colleagues also identified TP53 R175H, and to a lesser extent wild type TP53, interacted with both MCM2 and MCM4^48^ using co‐immunoprecipitation (co‐IP) in H1299 cells. Two additional TP53 mutants were shown to co‐localize with MCM2. Proximity ligation assay (PLA) was used to detect interactions between R280K and R248Q mutant TP53 and MCM2 in MDA‐MB‐231 and HCC70 cells, respectively,^48^ indicating these proteins co‐localize. Importantly, authors note that a lack of a strong co‐IP with MCM proteins but observe strong proximity (PLA) signal indicates the mutant TP53 and MCM “interaction is not due to a strong direct protein‐protein interaction”.^48^ They conclude that “all forms of TP53 can be found in close proximity to MCM proteins” and mutant TP53 proteins exhibit a stronger PLA signal.^48^

It is worth noting that the TP53 construct from this study contains two mutations (P72R and P278A). The P72R is a common mutation in 40% of many populations and is associated with some diseases but is predicted to be benign.^66^ At the same time, P278A is a less common mutation to the core domain of TP53. A recent study predicted the R278A mutation would likely be harmful due to changes in dynamic properties,^66^ but this was not confirmed with in vitro experiments, to the best of our knowledge. We cannot entirely discount the importance of the other MCM fragment pairs partnered with TP53 fragments that contain the P72R or P278A mutation without further quantitative and qualitative analysis comparing binding or function relative to wild type protein. What remains to be clarified is how the P278A mutant from this study ranks among other TP53 mutants when considering function (or protein activity) and substrate affinity. Nonetheless, wild type TP53 was found to interact with three MCM proteins which expands recent in vivo results giving evidence of a transient interaction between wild type TP53 and MCM2 and MCM4.^48^, ^50^

One explanation for the AVA‐Seq method being able to identify these interactions could be the increased sensitivity of the assay. By this, we mean if the binding affinity of wild type TP53 to MCM proteins is higher (weaker or more transient interaction) than the gain‐of‐function mutation R273H with a given substrate, there would be an increased challenge in detecting interactions. The power of AVA‐Seq is it allows us to look beyond the expected interactions to determine other exciting and potentially novel biologically relevant interactions that warrant further evaluation. It would be worth exploring the range of binding affinities detectable by AVA‐Seq by using proteins with known binding affinity and making a “standard curve” by increasing competitive inhibition ranging from 0 to 5 mM 3‐AT, for example.

In the future, we plan to screen wild type TP53 and a library TP53 mutants against the human genome, allowing researchers to select potentially subtle differences in protein–protein interactions to examine further. TP53 gain‐of‐function mutations can inherently change the pool of interacting proteins, resulting in different biological effects.^67^ Protein mutations can significantly impact protein interaction patterns. The AVA‐Seq method would allow the interaction space to be narrowed, providing a more targeted approach to drug screening. This would be advantageous because targeted inhibition of a particular cancer mutation would have minimal effect on normal cells. Identifying unique targets of TP53 mutants that do not bind to wild type TP53 could open pathways to new drug targets up and downstream, offering unique therapeutic opportunities to treat cancer.^68^, ^69^

In conclusion, we overlayed protein fragment interaction pairs with known protein contact points from a recent cryo‐EM structure of the MCM complex allowing us to obtain a high resolution view of the interacting domains. We show the intrinsically disordered wild type TP53 interacts with MCM2, MCM3, and MCM5. We postulate these interactions may not have been identified previously due to the weaker binding of a wild type TP53 compared to a gain‐of‐function TP53 mutant. Finally, we generated a mini‐cancer interactome highlighting known and novel protein interactions between TP53, MCM2, MCM3, MCM5, NOD1, SMAD1, SMAD4, ORC2, ORC4, and HSP90AA1. We believe these will provide an excellent starting point for potential novel drug design.

## CONFLICT OF INTEREST

The authors declare that there are no conflict or competing interests.

## AUTHOR CONTRIBUTIONS

S.S.‐R. conceived the idea and designed the study; S.S.‐R. and N.M.A. collected the data; S.S.‐R., J.A., and J.A.M. analyzed the data; S.S.‐R. and J.A.M. wrote the manuscript.

## FUNDING INFORMATION

This research was supported by funding from Qatar Foundation to Weill Cornell Medicine in Qatar in the form of the BMRP2 grant.

## ETHICAL APPROVAL STATEMENT

All analyses were based on previous studies; thus, no ethical approval and patient consent are required.

## Supporting information


Table S1–S5

## Data Availability

Sequences were deposited to the Sequence Read Archive of NCBI under the bioproject ID PRJNA756122.
