# Supplementary material for: Novel protein contact points among TP53 and minichromosome maintenance complex proteins 2, 3, and 5
Source: Cancer Med. 2022 May 14;11(24):4989–5000. doi: 10.1002/cam4.4805 (PMC9761056; doi:10.1002/cam4.4805)
Supplement: Supplementary file 1 — Table S1–S5 [file CAM4-11-4989-s001.docx]

*Novel protein contact points among TP53 and MCM complex proteins 2, 3, and 5*

Stephanie Schaefer-Ramadan^1*^, Jovana Aleksic^1^, Nayra M. Al-Thani^1^ and Joel A. Malek^1^

^1^Department of Genetic Medicine, Weill Cornell Medicine in Qatar, Doha 24144, Qatar.

* To whom correspondence should be addressed.

Tel: +974 4492 8465

Email: [SAR2038@qatar-med.cornell.edu](mailto:SAR2038@qatar-med.cornell.edu)

Supplemental Data

Supplemental Table 1 MCM2 and MCM5 interacting fragments.

| Protein 1: fragment start (a.a.) | Protein 2: fragment start (a.a.) | 2 mM 3-AT | 5 mM 3-AT |
| --- | --- | --- | --- |
| MCM5:524 | MCM2:665 | X |  |
| MCM5:520 | MCM2:658 | X |  |
| MCM5:524 | MCM2:658 | X |  |
| MCM5:254 | MCM2:658 | X |  |
| MCM5:520 | MCM2:665 | X |  |
| MCM5:18 | MCM2:632 | X | X |
| MCM5:18 | MCM2:658 | X | X |
| MCM5:207 | MCM2:80 | X |  |
| MCM5:212 | MCM2:80 | X |  |
| MCM2:467 | MCM5:93 |  | X |

Supplemental Table 2 MCM2 and MCM3 interacting fragments.

| Protein 1: fragment start (a.a.) | Protein 2: fragment start (a.a.) | 2 mM 3-AT | 5 mM 3-AT |
| --- | --- | --- | --- |
| MCM2:669 | MCM3:600 | X |  |
| MCM2:669 | MCM3:590 | X |  |
| MCM2:640 | MCM3:422 | X |  |
| MCM2:36 | MCM3:190 | X |  |
| MCM2:335 | MCM3:606 | X | X |
| MCM2:532 | MCM3:636 | X | X |
| MCM2:643 | MCM3:634 | X | X |
| MCM3:194 | MCM2:512 | X |  |
| MCM2:470 | MCM3:634 | X | X |
| MCM3:194 | MCM2:643 | X |  |
| MCM3:218 | MCM2:512 | X |  |
| MCM2:470 | MCM3:636 | X | X |
| MCM2:331 | MCM3:634 | X | X |
| MCM2:609 | MCM3:538 | X | X |
| MCM2:597 | MCM3:580 | X | X |
| MCM2:712 | MCM3:634 | X | X |
| MCM2:80 | MCM3:634 | X | X |
| MCM2:712 | MCM3:628 | X |  |
| MCM2:488 | MCM3:580 | X |  |
| MCM2:669 | MCM3:634 | X | X |
| MCM2:93 | MCM3:634 | X | X |
| MCM3:578 | MCM2:80 | X |  |
| MCM2:488 | MCM3:606 | X |  |
| MCM2:515 | MCM3:538 | X |  |
| MCM2:470 | MCM3:606 | X | X |
| MCM2:643 | MCM3:527 | X |  |
| MCM2:488 | MCM3:607 | X |  |
| MCM2:40 | MCM3:634 | X |  |
| MCM2:80 | MCM3:604 | X |  |
| MCM3:551 | MCM2:84 | X |  |
| MCM2:470 | MCM3:629 | X |  |
| MCM2:488 | MCM3:598 | X |  |
| MCM2:467 | MCM3:634 | X | X |
| MCM2:81 | MCM3:730 | X | X |
| MCM2:165 | MCM3:489 | X |  |
| MCM2:669 | MCM3:538 | X |  |
| MCM2:656 | MCM3:605 | X |  |
| MCM2:640 | MCM3:190 | X |  |
| MCM2:521 | MCM3:634 |  | X |
| MCM2:318 | MCM3:538 |  | X |
| MCM2:488 | MCM3:636 |  | X |
| MCM2:643 | MCM3:538 |  | X |
| MCM2:656 | MCM3:634 |  | X |
| MCM2:746 | MCM3:606 |  | X |
| MCM2:646 | MCM3:634 |  | X |
| MCM2:470 | MCM3:176 |  | X |

Supplemental Table 3 MCM3 and MCM5 interacting fragments.

| Protein 1: fragment start (a.a.) | Protein 2: fragment start (a.a.) | 2 mM 3-AT | 5 mM 3-AT |
| --- | --- | --- | --- |
| MCM5:520 | MCM3:422 | X |  |
| MCM5:520 | MCM3:190 | X |  |
| MCM3:168 | MCM5:93 | X | X |
| MCM3:488 | MCM5:82 | X |  |
| MCM5:524 | MCM3:190 | X |  |
| MCM5:18 | MCM3:190 | X |  |
| MCM3:598 | MCM5:20 | X |  |
| MCM5:18 | MCM3:176 | X |  |
| MCM5:520 | MCM3:175 | X |  |
| MCM5:35 | MCM3:634 | X | X |
| MCM5:85 | MCM3:634 | X | X |
| MCM5:18 | MCM3:628 | X |  |
| MCM5:254 | MCM3:184 | X |  |
| MCM5:85 | MCM3:636 | X | X |
| MCM5:86 | MCM3:634 | X | X |
| MCM5:254 | MCM3:538 | X | X |
| MCM5:14 | MCM3:538 | X |  |
| MCM5:88 | MCM3:184 | X |  |
| MCM5:86 | MCM3:188 | X |  |
| MCM5:190 | MCM3:634 |  | X |
| MCM5:188 | MCM3:636 |  | X |
| MCM5:103 | MCM3:636 |  | X |
| MCM5:103 | MCM3:538 |  | X |
| MCM5:88 | MCM3:606 |  | X |
| MCM5:296 | MCM3:517 |  | X |
| MCM5:86 | MCM3:636 |  | X |
| MCM5:127 | MCM3:634 |  | X |
| MCM5:254 | MCM3:634 |  | X |
| MCM3:225 | MCM5:57 |  | X |

Supplemental Table 4 TP53 and MCM2, MCM3, and MCM5 interacting fragments. * Represents TP53 fragments which are wildtype.

| Protein 1: fragment start (a.a.) | Protein 2: fragment start (a.a.) | 2 mM 3-AT | 5 mM 3-AT |
| --- | --- | --- | --- |
| *TP53:296 | MCM2:93 | X |  |
| TP53:185 | MCM2:515 | X |  |
| TP53:177 | MCM2:473 | X |  |
| TP53:220 | MCM2:515 | X |  |
| *TP53:89 | MCM2:512 | X |  |
| TP53:134 | MCM3:404 | X |  |
| *TP53:89 | MCM3:606 | X | X |
| TP53:271 | MCM3:634 | X | X |
| TP53:50 | MCM3:634 | X | X |
| *TP53:284 | MCM3:606 | X | X |
| TP53:217 | MCM3:634 | X | X |
| *TP53:89 | MCM3:628 | X |  |
| TP53:163 | MCM3:634 | X | X |
| *TP53:80 | MCM3:634 | X | X |
| *TP53:97 | MCM3:634 | X | X |
| *TP53:89 | MCM3:602 | X | X |
| MCM3:225 | TP53:135 | X |  |
| *TP53:89 | MCM3:580 | X | X |
| TP53:214 | MCM3:538 | X |  |
| TP53:271 | MCM3:636 |  | X |
| TP53:214 | MCM3:628 |  | X |
| MCM5:520 | TP53:220 | X |  |
| MCM5:454 | TP53:197 | X |  |
| MCM5:324 | TP53:135 | X |  |
| *TP53:89 | MCM5:4 | X | X |
| MCM5:222 | *TP53:106 | X |  |
| MCM5:127 | TP53:186 |  | X |

Supplemental Table 5. Interactions between wildtype or mutant TP53 and proteins of the MCM complex.

| **TP53 construct** | **Interacting partner** | **System** | **Method** | **Reference** |
| --- | --- | --- | --- | --- |
| Wildtype TP53 | MCM2, MCM4 | H1299 | Co-IP | ^1^ |
| Wildtype TP53 | MCM2 | MCF7 | PLA | ^1^ |
| Wildtype TP53 | MCM2 | HCT116, MDA-MB-468 | PLA | ^2^ |
| TP53 R172H | MCM7 | thymic lymphomas from Trp53^R172H/R172H^ mice | IP followed by LC-MS/MS | ^3^ |
| TP53 R172H | MCM4 | thymic lymphomas from Trp53^R172H/R172H^ mice | Co-IP | ^1^ |
| TP53 R175H | MCM2, MCM4 | H1299 | Co-IP | ^1^ |
| TP53 R273H | MCM2 | MDA-MB-468 | PLA | ^1^ |
| TP53 R273H | MCM2, MCM4, MCM7 | MDA-MB-468, HT-29 | Western blot | ^1^ |
| TP53 R273H | MCM 2-7 |  | Proteomic enrichment analysis | ^1^ |
| TP53 R273H | MCM4 | MDA-231.shp53, HT-29 | Western blot | ^4^ |
| TP53 R273H | MCM2 | MDA-MB-468 | Co-IP | ^5^ |
| TP53 R273H | MCM7 | MDA-MB-468 | Co-IP | ^5^ |
| TP53 R273H | MCM2 | HCT116 | PLA | ^2^ |
| TP53 R273H Δ381-388 | MCM2 | MDA-MB-468 | PLA | ^2^ |
| TP53 R273H R337C | MCM2 | HCT116 | PLA | ^2^ |
| TP53 R273H A347D | MCM2 | HCT116 | PLA | ^2^ |
| TP53 R273H L344P | MCM2 | HCT116 | PLA | ^2^ |
| TP53 R280K | MCM2 | MDA-MB-231 | PLA | ^1^ |
| TP53 R248Q | MCM2 | HCC70 cells | PLA | ^1^ |

1. Qiu, W.-G. *et al.* Identification, validation, and targeting of the mutant p53-PARP-MCM chromatin axis in triple negative breast cancer. *NPJ breast cancer* **3**, 1 (2017).

2. Annor, G. K. *et al.* Oligomerization of Mutant p53 R273H is not Required for Gain-of-Function Chromatin Associated Activities. *Frontiers in Cell and Developmental Biology* **9**, (2021).

3. Yue, X. *et al.* BAG2 promotes tumorigenesis through enhancing mutant p53 protein levels and function. *eLife* **4**, (2015).

4. Polotskaia, A. *et al.* Proteome-wide analysis of mutant p53 targets in breast cancer identifies new levels of gain-of-function that influence PARP, PCNA, and MCM4. *Proceedings of the National Academy of Sciences* **112**, E1220–E1229 (2015).

5. Xiao, G. *et al.* Gain-of-Function Mutant p53 R273H Interacts with Replicating DNA and PARP1 in Breast Cancer. *Cancer Research* **80**, 394–405 (2020).
